# Current and Future Role of Tyrosine Kinases Inhibition in Thyroid Cancer: From Biology to Therapy

**DOI:** 10.3390/ijms21144951

**Published:** 2020-07-13

**Authors:** María San Román Gil, Javier Pozas, Javier Molina-Cerrillo, Joaquín Gómez, Héctor Pian, Miguel Pozas, Alfredo Carrato, Enrique Grande, Teresa Alonso-Gordoa

**Affiliations:** 1 Medical Oncology Department, Hospital Universitario Ramón y Cajal, 28034 Madrid, Spain; mariasanro21@gmail.com (M.S.R.G.); pozas.javier@gmail.com (J.P.); pozas.miguel12@gmail.com (M.P.); acarrato@telefonica.net (A.C.); talonso@oncologiahrc.com (T.A.-G.); 2 The Ramon y Cajal Health Research Institute (IRYCIS), CIBERONC, 28034 Madrid, Spain; 3 Medicine School, Alcalá University, 28805 Madrid, Spain; jgomezramirez@hotmail.com (J.G.); hectorpian@yahoo.es (H.P.); 4 General Surgery Department, Hospital Universitario Ramón y Cajal, 28034 Madrid, Spain; 5 Pathology Department, Hospital Universitario Ramón y Cajal, 28034 Madrid, Spain; 6 Medical Oncology Department, MD Anderson Cancer Center, 28033 Madrid, Spain; egrande@oncomdrid.com

**Keywords:** thyroid cancer, tyrosine kinase inhibitors, immunotherapy, RET, NTRK

## Abstract

Thyroid cancer represents a heterogenous disease whose incidence has increased in the last decades. Although three main different subtypes have been described, molecular characterization is progressively being included in the diagnostic and therapeutic algorithm of these patients. In fact, thyroid cancer is a landmark in the oncological approach to solid tumors as it harbors key genetic alterations driving tumor progression that have been demonstrated to be potential actionable targets. Within this promising and rapid changing scenario, current efforts are directed to improve tumor characterization for an accurate guidance in the therapeutic management. In this sense, it is strongly recommended to perform tissue genotyping to patients that are going to be considered for systemic therapy in order to select the adequate treatment, according to recent clinical trials data. Overall, the aim of this article is to provide a comprehensive review on the molecular biology of thyroid cancer focusing on the key role of tyrosine kinases. Additionally, from a clinical point of view, we provide a thorough perspective, current and future, in the treatment landscape of this tumor.

## 1. Introduction

### 1.1. General Aspects

The incidence of thyroid cancer has been steadily increasing over the past decades, mainly due to the evolution of diagnostic techniques and clinical surveillance. However, mortality from thyroid cancer has remained relatively stable over time [1]. Nowadays, one of the main challenges faced by clinicians is how to balance the therapeutic approach to avoid overtreatment in patients with an indolent tumors, while providing adequate care to patients with a more advanced or aggressive disease.

Thyroid cancer represents a heterogeneous neoplasm, with distinct histologic subtypes, resulting in different molecular pathways, clinical presentation, therapeutic approaches and prognosis. However, all thyroid cancer subtypes share common features related to the activation of particular molecular pathways that involve different tyrosine kinases. These molecules have been studied as therapeutic targets with successful results in randomized clinical trials. The aim of this article is to provide a comprehensive review on the molecular biology of thyroid cancer focusing on the key role of tyrosine kinases and the future perspective in the therapeutic algorithm of these tumors.

### 1.2. Histological Subtypes

Thyroid cancer can be histologically classified into three main subtypes: differentiated thyroid cancer (DTC; including papillary, follicular, Hürthle and poorly differentiated thyroid cancer), anaplastic thyroid cancer (ATC) and medullary thyroid cancer (MTC) [2]. Recently, the WHO has published an updated classification of thyroid neoplasms with several histological nuances according to the advancement in the knowledge of novel genetic and molecular particularities [3].

#### 1.2.1. Follicular-Derived Thyroid Cancers

Differentiated Thyroid Cancer (DTC) accounts for over 97% of all cases of thyroid cancer [1]. In general, they have an excellent prognosis with a 5-year overall survival (OS) rate above 95%. However, patients diagnosed in stages III-IV have a worse outcome, with a 10-year survival rate < 50–60% [2].

Papillary thyroid cancer (PTC) is the most prevalent subtype, representing 80% of all thyroid cancers. PTC has the best prognosis with a 5-year survival rate of 97.7%. In terms of histopathology, PTC includes classic forms (cPTC) and variant forms, which comprises follicular variant, tall cell variant and several other variant types, which are all rarely found [2].

Follicular thyroid cancer (FTC) constitutes 10–20% of all DTC, with a higher prevalence in iodine-deficient geographical areas. It is important to underline that, even though they also have a good prognosis, up to 5–15% of patients will die because of their disease, since minimally invasive tumors tend to have microscopically foci of capsular invasion, which leads to a slightly worse prognosis [2].

Hürthle cell carcinoma accounts for 2–8% of DTC and is composed by eosinophilic thyroid cells with a large number of abnormal mitochondria. The prognosis worsens when there is a lower iodine uptake and the capsular and/or vascular invasion [2].

Recently, the poorly differentiated thyroid cancer (PDTC) has been characterized as a new form of DTC, being the most aggressive subtype, with a mean survival of 3.2 years. The diagnostic criteria for this tumor is based on the Turin proposal: invasive tumors with a solid/trabecular/insular growth and presence of a mitotic index > 3 per 10 high-power fields, necrosis or convoluted nuclei [2].

Anaplastic Thyroid Cancer represents approximately 0.8% of all thyroid cancers diagnosis [1], either arising from a previously developed DTC or appearing de novo. Despite having a very poor prognosis, with a 5-year overall survival rate of 12%, promising preliminary results from ongoing clinical trials may shed light on this aggressive subtype. Three histological subtypes of anaplastic thyroid cancer (ATC) have been described: sarcomatoid (composed of malignant spindle cells), giant cell (abundant in pleomporphic cells) and epithelial (harboring squamoid or squamous tumor nests). Other relevant histological findings comprise a high proliferative rate, marked pleomorphism and extensive vascular invasion [3].

#### 1.2.2. Neuroendocrine C-Cell-Derived Thyroid Cancers

Medullary thyroid cancer (MTC) accounts for up to 1.6% of thyroid cancers [1]. MTC is a very heterogeneous neoplasm which arises from the parafollicular cells (C cells), that produce calcitonin. In 25% of patients, MTC is part of an inherited syndrome: familial MTC or the multiple endocrine neoplasia syndromes type 2A and 2B (MEN2A and MEN2B). In the remaining 75% of cases, it arises sporadically (sMTC). Somewhere in between 20 and 40% of patients with MTC will, unfortunately, develop metastasis leading to a 10-year survival rate of 40%.

## 2. Molecular Biology

### 2.1. Differentiated TC (TCGA)

#### 2.1.1. Papillary Thyroid Cancer

The Cancer Genome Atlas (TCGA) published in 2014 a pan-genomic analysis of the largest cohort of PTCs (*n* = 496), excluding poorly differentiated and undifferentiated carcinomas. TCGA discovered new genetic alterations in previously known oncogenic drivers, as well as new drivers, such as *EIF1AX*, *PPM1D* and *CHEK2*, reducing the amount of PTC of unknown origin, that dropped from 25% to 3.5% [4].

Seventy to 80% of the genomic aberrations found in PTC are due to rearrangements of *RET/NTRK* and activating mutations of *BRAF* and *RAS* that lead to the activation of the mitogen-activated protein kinase (MAPK) pathway, and, hence, promote tumorigenesis. These two main groups of genetic alterations are mutually exclusive. Also, *ALK* rearrangements, and *EIF1AX* and *TERT* mutations are additional drivers. In general, PTCs have one of the lowest tumor mutational burden, usually carrying a single driver, which may explain their frequent indolent behavior. Nonetheless, 9% of cases express both *TERT* and *BRAF/RAS* mutations, resulting in a worse outcomes. These genetic alterations are conceived as strong drivers with the exception of *RAS* mutations since they are commonly found in benign thyroid neoplasms [5,6].

TCGA divided PTCs into two major subtypes: *BRAFV600E*-like (BVL) and *RAS*-like (RL), representing a molecular classification instead of the classic histopathological one. BVL is associated with various fusion genes, such as *RET* and *NTRK1/3*, whereas RL is related to *EIF1AX* and fusion genes, such as *PAX8-PPARγ-1*. A third subdivision of tumors was later proposed: non-*BRAF*-non-*RAS* (NBNR), which is associated with a more indolent behavior [4,7].

*RET* and *NTRK* rearrangements: *RET* and *NTRK1* genes code for transmembrane tyrosine kinases which are usually not expressed in thyroid cells. Because of the rearrangement, a chimeric gene is formed resulting in MAPK-signaling pathway activation. In the case of *RET*, its chimeric gene is *RET/PTC* and in the case of *NTRK*, it is *TRK*. They have been identified in 20–40% of PTC, with a predominance of RET/PTC over TRK (threefold more frequent) [8]. Different chimeric *RET* and *TRK* genes have been identified, harboring a similar prognosis. The most common *RET* rearrangements in PTC (≈90%) are *CCDC6-RET (RET/PTC1)* (59%) and *NCOA4-RET (RET/PTC3)* (36%). They are usually found in patients with previous ionizing radiation exposure (70% of Chernobyl survivors’ cancers) and children. They are related to microcarcinomas, multifocal PTC and confer an unfavorable disease presentation and outcome [9].

*RAF*, a cytoplasmic serine–threonine protein kinase is essential in the activation of the MAPK pathway. Approximately 40–60% of PTC harbor a *BRAF* mutation, being *BRAF T1799A* transversion resulting in *BRAFV600E*, the most prevalent one (95%), with an almost 500-fold increase in kinase activity. *BRAF* mutations are involved only in the development of PTC and ATC, with no evidence of activity in adenomas, MTC or other types of DTC [10]. It is associated with tumor growth, lymph node metastases, advanced locoregional stage at initial surgery and lower expression of genes involved in iodine metabolism [11]. Interestingly, *BRAF* mutation may appear in lymph node metastases with no expression in the primary tumor [12]. All these data confer a poor prognosis even in small PTC [13]. It has recently been reported that age and male sex are independent risk factors of poor outcome in *BRAF*-mutated PTC [14].

In the case of *RAS* oncogenes, activating mutations in codons 12, 13 and 61 of the three *RAS* genes (*NRAS, KRAS* and *HRAS* mutations) are found in 4.01%, 1.54% and 0.31% of PTC, respectively. However, they are most commonly found in FTC (40%) and in follicular variant PTC (FV-PTC). Similarly to *BRAF* mutations, they activate MAPK-signaling pathways. In addition, *RAS* alterations also trigger PI3K/AKT intracellular signaling, resulting in a higher expression of iodine-related genes [15,16].

Interestingly, FV-PTC, which shares the follicular growth pattern with the FTC and nuclear features of PTC, show an intermediate mutational status between FTC and cPTC. Just like in FTC, *RAS* genetic alterations are common. However, BRAF mutations, which are scarce in FTC, can be found in FV-PTC. Moreover, follicular-patterned thyroid tumors frequently present an isolated deletion of chromosome 22q. *NF2* and *CHEK2* tumor suppressor genes are located in this location. Among FV-PTC, a further subclassification has been proposed: encapsulated (EFV-PTC) and infiltrative neoplasm, with a molecular similarity with FA/FTC and classic PTC, respectively. Moreover, EFV-PTC can be divided into invasive EFV-PTC and non-invasive follicular thyroid neoplasm with papillary-like nuclear features (NIFTP), with a higher *BRAFV600E* and *RAS* mutation rate, respectively [4,17].

*TERT* promoter mutations are found in 7.5% of PTC and 17.1% of FTC and are associated with tumor dedifferentiation from DTC to PDTC (29%) or ATC (33.3%). Its aggressive behavior is enhanced by a co-mutation with *BRAFV600E*. Notwithstanding, its coexistence with *RAS* mutations in FTC has not been studied to date [18,19].

#### 2.1.2. Follicular Thyroid Cancer

In 2016, Song YS et al. analyzed the transcriptional and mutational landscape of follicular adenoma (FA), minimally-invasive FTC (miFTC), FV-PTC, as well as PTC. FA and miFTC expressed a similar mutational profile, with H/K/NRAS genetic alterations in up to 40% of tumors, followed by *DICER1, EIF1AX, EZH1, SPOP, IDHI, SOS1* and *PAX8-PPARγ-1* mutations, being all of them exclusive with each other. The presence of these other alterations suggest that different pathways apart from PI3K/AKT or MAPK are involved in the FA/FTC tumorigenesis [7]. These findings were subsequently confirmed by other studies [20].

As previously mentioned, point mutations in *RAS* genes are found in up to 40% of FTC, with a predominance of *N-RAS*. However, *K-RAS* activation is commonly described in radiation induced follicular cancers. They are associated with poor histological differentiation and survival, as well as a higher risk of dedifferentiating into ATC [21]. *PAX8-PPARγ-1* rearrangement is found in FA and FTC with a prevalence of 10 and 41%, respectively. Its functional consequences are not clear, but it has been suggested to be a marker of vascular invasion [22].

*EIF1AX* codes for eukaryotic translation initiation factor 1A (eIF1A), playing a role in the recognition of the start codon in the translation process. Mutations in this gene are found with a higher frequency in follicular patterned carcinomas and adenomas: 5.45% in FTC and FA followed by 3% in FV-PTC and 0.62% in cPTC [4].

Additionally, higher copy number variations (CNVs) have been described in FTC and FVPTC in comparison with cPTC. In the case of FA, they show a lower percentage of CNVs supporting its preneoplastic condition despite sharing a similar genetic profile with FTC. Nonetheless, there is still no definitive molecular marker to properly distinguish FA from FTC [23].

Concerning the expression of micro-RNAs (miRNAs), the TCGA confirmed the upregulation of miR-221, miR-222 and miR-146 in PTC, which play a role in the aggressiveness of the tumor. In the case of FTC, a greater expression of miR-192, miR-197, miR-328, and miR-346 has been observed [24,25].

#### 2.1.3. Hürthle Thyroid Cancer

This tumor usually presents alterations in mitochondrial DNA (mtDNA), along with other mutations, such as *TERT* promoter (59%), *NRAS* (9%) and *KRAS* (6%) mutations. MtDNA alterations are found in both adenomas and carcinomas. Other frequently found aberrations are the trisomy of chromosomes 7 and 12, and mutations of GRIM-19 (Gene associated with retinoid-interferon-b-induced mortality), which regulates mitochondrial functions [26,27].

#### 2.1.4. Poorly Differentiated Thyroid Cancer

Landa et al. performed a genomic sequencing of 84 PDTC and 33 ATC suggesting that both of them could arise from DTC through the accumulation of additional aberrations from an original common precursor. A higher mutation burden is associated with older age, larger tumors, presence of distant metastasis and a shorter survival. The prevalence of *BRAFV600E* mutations was 33% and of RAS mutations was 28%, being again mutually exclusive. *EIF1AX* appears in 11% of PDTCs, being strongly associated with *RAS* mutations, which differs from DTC where they are mutually exclusive. *TERT* promoter mutations were identified in 40% of PDTCs, in contrast with 7.5% in PTC. They may co-occur with *BRAF/RAS* mutations, conferring more aggressiveness. Chromosomal rearrangements, such as *RET/PTC, STRN-ALK* fusion or *PAX8-PPARG*, were observed in 14% of PDTC, and were associated with younger age [28].

### 2.2. Medullary Thyroid Cancer

MTCs have a rich genetic landscape and achieving a detailed genetic understanding may lead to the discovery of new biomarkers and potential targeted therapies. *RET* and *RAS* proto-oncogene aberrations are detected in approximately 90% of MTCs and are considered their main driver mutations [29].

*RET* activation induces downstream pathways that promote cell growth, proliferation, survival and differentiation, including the MAPK and PI3K signaling pathways. The activation of the *RET* receptor occurs after the glial cell-line derived neurotrophic factor (GDNF) family of ligands (GFLs) bind to GDNF family receptor-α (GFRα), forming the GFL/GFRα complex and allowing the dimerization of the receptor, which results in the phosphorylation of specific tyrosine residues within the tyrosine kinase domain of the *RET* receptor [30]. Genetic alterations involving the *RET* proto-oncogene have been typically described in MTCs and PTCs. MTC mainly contains point mutations and few deletions and insertions, whereas in PTCs only chromosomal arrangements are found [31].

#### 2.2.1. Germ Line *RET* (*Rearranged during Transfection*) Mutations

The association between germline *RET* mutations and *MEN2* was identified over two decades ago. It is known that the MEN2A phenotype is linked to mutations in *RET* cysteine codons located in exons 10 and 11, resulting in ligand-independent constitutive activation of the tyrosine kinase receptor by the formation of disulfide-bonded homodimers. On the other hand, the MEN2B phenotype is almost exclusively associated with the Met918Thr (M918T) mutation in exon 16, leading to a constitutive high level of phosphorylation. In both cases, the final effect consists on the upregulation of the MAPK and PI3K signaling pathways, thus causing uncontrolled cell growth and proliferation [32].

#### 2.2.2. Somatic *RET* (*Rearranged during Transfection*) Mutations

Roughly 66% of MTCs present somatic mutations in *RET* [33]. This is associated with lymph node metastasis at diagnosis, lower biochemical cure rate after surgery and worse prognosis [34,35]. The point mutation M918T is the most common somatic *RET* mutation in sMTC (70.5% of all *RET* mutations), which also happens to be the most frequently found germline mutation in MEN2B [36]. The somatic M918T mutation, along with the rarer Ala883Phe (A883F) mutation, predict worse outcome with regard to other *RET* mutations or none [37]. Less frequently reported *RET* mutations is C634R (4.9%), among others [38,39]. Also, patients with a higher variant allele frequency (VAF) of the *RET* mutation have a worse prognosis [40]. M918T-mutated tumor DNA seems to be detectable in peripheral blood and, interestingly, preliminary evidence suggests that circulating M918T-mutated DNA can predict outcome more accurately than the calcitonin doubling time [41].

#### 2.2.3. Somatic RAS Mutations

Somatic *RAS* mutations are found in 10–45% of patients with sMTC (more commonly *HRAS* and *KRAS*) and in 80% of *RET*-negative sMTC, being most of the times mutually exclusive with *RET* genetic alterations [42]. As opposed to FTCs, sMTC tend to harbor *HRAS* mutations, being the p.Gln61Arg mutation the most frequently reported. Although there is no robust evidence linking *RAS* mutations and tumor behavior or prognosis, there are some data suggesting *RAS*-mutated sMTC may have a less aggressive clinical course than *RET*-mutated sMTC. For instance, a cohort of patients with sMTC was divided into four subgroups: high-risk somatic *RET* mutations (M918T and A883F), other *RET* mutations, *RAS* mutations and no mutations. They found that *RAS*-mutated tumors had a better clinical outcome when compared to high-risk *RET*-mutated tumors, but a worse prognosis than tumors with other *RET* mutations [43]. A recent analysis of 181 sMTC showed that *RAS*-mutant tumors had better outcome and a lower tumor staging than *RET*-mutant tumors [40].

#### 2.2.4. Other Pathways

In around 40% of patients, no *RAS* or *RET* proto-oncogene mutation is identified. The CDKN gene family has lately drowned out interest in the field of sMTC, since the somatic copy number loss in *CDKN2C* seems to be associated with worse overall survival (OS) [44] and could potentially become a therapeutic target [45]. Also, single nucleotide polymorphisms (SNPs) in the *CDKN1B* and *CDKN2A* genes, increase the susceptibility to sMTC [46]. Furthermore, recent analysis identified loss of heterozygosity in *CDKN2C* and *CDKN2D*, which can become a potential prognostic factor [47].

The mTOR pathway is activated in *RAS*-mutant sMTC and it seems that a high mTOR activity measured by pS6 expression is associated with a worse clinical outcome [48].

The heterozygous loss of retinoblastoma (RB) and other components of the RB pathway, including cyclin-dependent kinase (CDK) inhibitors p18 and p27, are involved in MTC tumorigenesis in murine models and in human tumor samples [49,50]. Also, it seems that a reduced RB protein expression is associated with lower survival [51]. Recently, CDK1/2/5/9 inhibitor dinaciclib proved to lower CDK7, RET protein and mRNA levels in human MTC cells, showing a synergistic effect when combined with a RET kinase inhibitor [52].

Overexpression of vascular endothelial growth factor (VEGF)-2 receptors has been reported in MTC [53] and is associated with an increased risk of developing distant metastasis [54]. Mutations in *MET* and fibroblast growth factor receptor (FGFR) have also been described in MTC [55]; the inhibition of the latter has the ability to decrease the proliferation of MTC cells [56].

In recent years, the genomic landscape of MTC has been thoroughly studied by NGS techniques. However most genetic alterations seemed to be anecdotic rather than significantly recurrent mutations [57,58].

### 2.3. Anaplastic Thyroid Cancer

The mutational landscape of ATC is very heterogeneous, holding a greater mutation burden than DTC and PDTC. According to an NGS analysis of 196 ATC, the two most commonly mutated genes are *TP53* (65%) and *TERT* (65%). *BRAF* mutations are found in 41% of ATCs and 27% of them harbour RAS genetic alterations. Apart from *TP53*, other tumor suppressor genes are sometimes mutated in ATC, mainly *NF2, RB1* and *NF1*. Mutations in histone methyltransferases (HMT) functional groups and in the chromatin remodeling complex SWI/SNF have also been described in approximately 24% and 19% of ATCs respectively, being significantly more frequent than in DTCs. Mutations in the PI3K/AKT/mTOR signaling pathway are also abundant within ATCs. Alterations in cell cycle regulators are found in around 29% of the cases, affecting genes such as *CDKN2A, CDKN2B* and *CCNE1. KIT* amplification and co-amplification of tumor immune evasion genes (PD-L1, PD-L2 and JAK2) seems to be specific to ATCs, not being found in other tumor subtypes. Mutations in other genes, including mTOR pathway modulator INPP4B, transcription factor NFE2L2, DNA repair gene NBN, caspase CASP8 and a member of ephrin receptor subfamily EPHA3, although rare, were significantly associated with ATC [28,59].

When it comes to driver mutations, *BRAF* mutations are less prevalent with respect to DTC, whereas *RAS* mutations are more frequent. Among *BRAF* mutations, *BRAFV600E* is the most common. Mutations in *BRAF* are mutually exclusive with mutations in *KRAS, NRAS or HRAS*. Similar to PTC, a micro-RNA array analysis of *BRAF*-mutated ATC showed they belong to the *BRAF*-like category. Interestingly, *RAS*-mutated ATC lose the genetic characteristics exhibited in *RAS*-mutated DTC, being hence classified as *BRAF*-like tumors. This indicates that regardless of the driver mutation, a high transcriptional MAPK pathway activity is characteristic of ATCs [28]. *BRAF*-mutant ATCs also show an upregulation of VEGF and notch-signaling pathways. *RAS*-positive ATCs harbor an upregulation of focal adhesion, extracellular matrix (ECM) receptor interaction, p53-signaling pathway, cell cycle, classically-activated macrophages (CAMs) and MAPK signaling pathway with respect to other subtypes of TC. Particularly, the JAK-STAT-signaling pathway was activated in this subset of ATCs, and JAK inhibition with ruxolitinib decreased the expression of the JAK-STAT pathway downstream molecules (SOCS3, BCL2L1, and MYC) [60]. Co-mutations in AKT1/PIK3CA or EIF1AX were frequently found in *BRAF* or *RAS*-mutated ATCs, which seems to indicate that the progression to ATC can be predicted by different markers depending on the driver mutation of DTCs [60].

Tumor suppressor genes alterations have also been described. *TP53* mutations are highly prevalent in ATCs and can be used to distinguish ATCs from other subtypes of TC. NF1 mutations have been found in three *BRAF/RAS* WT ATCs, the three of them showed truncating alterations in *PTEN* [28]. Along with *PTEN* mutations, genetic abnormalities in other key effectors in the *PI3K/AKT* are characteristic of ATCs. For instance, *PIK3CA* gene mutations tend to co-occur with *BRAF* mutations [59]. *EIF1AX* encodes for a key component of the translation preinitiation complex (PIC) [61]. Mutations in *EIF1AX* appear in approximately 9% of ATCs and are strongly associated with *RAS* mutations, which contrasts with PTC, where these mutations are mutually exclusive. Also, *EIF1AX* mutations do not co-exist with mutations in the PI3K/AKT pathway [28].

As opposed to PTC, in which *TERT* promoter mutations are only found in 7.5% of tumors, 73% of ATCs hold *TERT* genetic alterations and they tend to co-occur with *BRAF* and *RAS* mutations, harbouring a worse prognosis [62]. Interestingly, *TERT* and *EF1AX* mutations do not overlap in *RAS*-mutant ATCs [28].

Loss-of-function mutations in cell cycle regulators CDKN2A and CDKN2B are more frequent in ATCs with respect to DTCs suggesting they play a key role in anaplastic transformation, making it a potential prognostic factor in DTCs. ATCs with CDKN2A loss are associated with an increased disease-specific mortality [60].

ATCs tend to harbour a high mutational burden, mainly through two mechanisms: impaired MMR and activity of APOBEC family of cytidine deaminases. MMR deficiency through loss-of-function mutations seems not to be associated with genetic alterations in *RAS, BRAF* or *RET*. The APOBEC signature, which was only found in *BRAF*-mutant ATCs, is associated with the progression of ATC [59]. Targeting APOBEC mutagenesis may be a therapeutic approach in the near future.

Furthermore, four genetically distinct types of ATC have been recently proposed. Cluster 1 which includes *BRAFV600E*-mutated tumors, being associated with mutations in the mTOR pathway and resembling PTC, thus probably somehow deriving from them. Cluster 2 comprises ATC with genetic alterations in cell cycle regulators such as CDKN2A and CDKN2B, but also carrying genetic features of the other three clusters (*BRAF, NRAS* and *PTEN/NF1/RB1* mutations). Cluster 3 ATC are believed to develop from *NRAS*-mutant FTCs since *NRAS* mutations are frequent in this category. Finally, cluster 4 includes ATC with oncogenic mutations in *RAS* genes, carrying *PTEN* mutations often coexisting with *NF1* and *RB1* mutations. Also, tumors belonging to cluster 4 have a higher mutational burden with genetic alterations in MSH2 and MLH1 [59].

## 3. From Molecular Knowledge to Clinical Benefit: Approved Drugs

### 3.1. Differentiated Thyroid Cancer

In spite of the excellent prognosis of DTC, locoregional recurrence appears in up to 20%, with 10% of patients developing distant metastasis in 10 years from diagnosis. The treatment of most patients with metastatic DTC includes surgery and selective use of radioactive iodine (RAI) ablation with thyroid stimulating hormone (TSH)-suppressive therapy according to risk assessment. TKI are considered for patients with progressive or symptomatic disease [63,64,65].

In general, RAI refractoriness has an incidence below 5% in patients with locorregional DTC, but almost 50% of metastatic DTC reach this clinical situation. This implies a worse outcome, with a 3-year OS below 50%. The criteria to define a RAI-refractory (RAI-R) DTC include the absence of RAI uptake at initial diagnosis of locoregional recurrence or metastasis, an absence or progressive reduction of RAI uptake after several doses, discrepancy in RAI uptake among different lesions in the post-therapy scan and tumor progression in 12 to 16 months even with previous RAI uptake in the post-therapy scan. Other criteria comprise an important uptake in 18FDG-PET, a total dose of RAI > 600 mCi, aggressive DTC histologies or unresectable primary tumors [65,66].

In this setting, watchful waiting can be considered in patients with asymptomatic disease, low tumor burden and stable or slowly progressive disease that is unlikely to spread rapidly or to be life-threatening. However, patients with progressive (assessed by RECIST v1.1) and/or symptomatic disease, are candidates for TKI initiation [65,66,67]. Nevertheless, it is not always easy to establish the time frame for RECIST 1.1 to define rapidly progressive disease. Hence, some approaches have been suggested for an accurate assessment on the optimal time for TKI initiation, such as the lung metastasis volume doubling time [68].

Tyrosine kinases are involved in the MAPK signaling pathway through phosphorylation/dephosphorylation of intracellular proteins. Lenvatinib and sorafenib have proved their efficacy in randomized phase III trials and become the standard of care in patients with DTC, based on the SELECT and DECISION trial, respectively. The main data coming from both phase III trials are summarized in Table 1.

Other multi-TKI (MKI) are currently being evaluated under phase III clinical trials to eventually reach the approval in this setting, such as cabozantinib, donafenib and apatinib. Vandetanib has also reached a phase III trial (VERIFY; NCT 01876784), but failed to meet the primary endpoint of PFS (10 months in the vandetanib arm vs. 5.7 months in the placebo arm; *p* = 0.08). Though more awaited in ATC, focus on BRAF inhibition has also been considered in patients with DTC harboring BRAFV600E mutations, achieving an ORR between 30–40%, approximately. This signaling inhibition has been suggested to have a potential role in RAI resensitization and is in line with the key relevance of tumor genotyping in thyroid cancer (refer to 4.5) [72]. Other drugs against different actionable targets are also under research and are summarized in Table 2.

### 3.2. Medullary Thyroid Cancer

Before the era of targeted therapy, systemic cytotoxic chemotherapy was the standard of care in patients with MTC, offering very poor outcomes hardly reaching a response rate of 20% [64,81,82].

Since *RET* proto-oncogene is mutated in most MTC, several clinical trials testing TKIs with *RET* inhibition activity have been carried out in the past decade. Initially, multikinase inhibitors (MKIs) such as cabozantinib and vandetinib, became the first-line systemic therapy for progressive metastatic MTC after being approved by the regulatory agencies based on their ability to improve survival in phase III randomized clinical trials [83,84] (Table 3).

**Figure 1:**
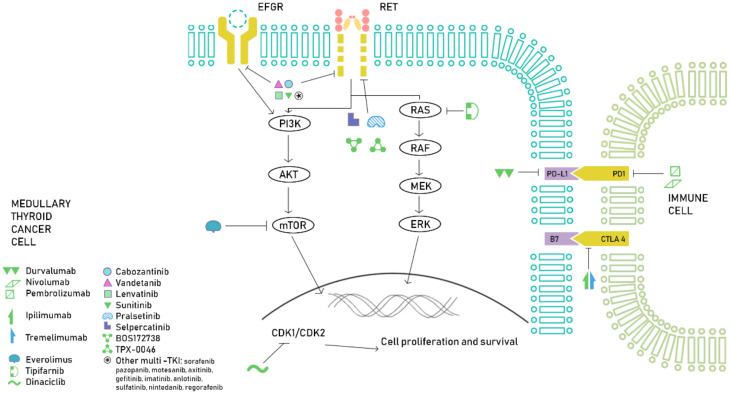
Overview of the current treatment approach in MTC. In this figure, we illustrate several drugs targeting the main molecular pathways involved in MTC tumorigenesis (MAPK and PI3K/AKT/mTOR), with special focus on the latest advances in *RET* inhibition.

Other MKIs have undergone phase II clinical trials, showing interesting results. Patients treated with sunitinib had a median PFS and OS of 16.5 and 29.5 months, respectively [85]. Lenvatinib reached a 6-months PFS of 67.5% and an ORR of 36% [86]. Other MKIs such as sorafenib or pazopanib have also shown activity in MTC [87,88]. Motesanib, axitinib, imatinib or gefitinib, have shown promising results in preclinical studies and phase I clinical trials, but failed to show enough efficacy to be used in daily clinical practice (Figure 1).

Since *RAS* mutations are frequently found in MTC, there is a growing interest in finding druggable targets in this setting. Farnesyl transferase inhibitors (FTI) prevents farnesylation of *RAS* proteins, blocking signal transduction and, thus, cessation of cell growth [89]. FTI tipifarnib is being investigated in a phase II clinical trial in *HRAS*-mutated tumors, including MTC [90].

### 3.3. Anaplastic Thyroid Cancer

**Figure 2:**
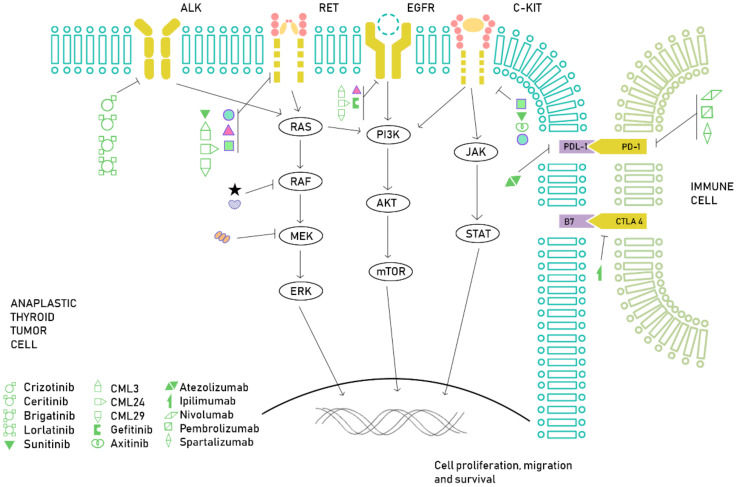
Overview of the current treatment approach in ATC. In this figure, we illustrate several drugs targeting the main molecular pathways involved in ATC tumorigenesis, with special focus on BRAF inhibition and immune checkpoint inhibitors.

As we stated before, ATC is a very aggressive tumor, often metastatic at diagnosis, with a poor prognosis even with a multimodal approach. In light of a better understanding of the genetic landscape of ATCs, several novel therapeutic approaches have been proposed, mainly in the field of targeted therapy and immunotherapy. For instance, *BRAFV600E* mutation is found in approximately 25% of patients with ATC and the combination of *BRAF* and *MEK* inhibitors, dabrafenib and trametinib, has been recently approved by the FDA for this subset of patients based on a recent phase II, open-label basket trial. This study included patients with *BRAFV600E*-mutated neoplasms, including 16 ATCs, reaching an ORR of 69% [91]. Enrollment in clinical trials is strongly advised, since the traditional cytotoxic chemotherapy is associated with very low response rates and a significant toxicity [92]. Several mutations and genomic aberrations, although rare, seem to be potentially targetable, such as *ALK* rearrangements, *NTRK* fusions, *TSC2* mutations or *RET* rearrangements [93,94], which along with a growing interest in the study of the immune tumor microenvironment, are leading several clinical studies that may have a significant impact in the management of this aggressive subtype of thyroid cancer (Figure 2).

A Japanese single-arm, open-label phase II clinical trial with lenvatinib was conducted in 51 patients, 17 of them with ATC, showed a 24% of objective response rate with an mPFS of 7.4 months and an OS of 10.6 months in the ATC subgroup of patients. These results allowed the approval of lenvatinib by the Japanese Regulatory Agency [95].

Several novel therapies involving TKIs are currently under research. Targeting angiogenesis has been one of the leading approaches as some *VEGFR* inhibitors, such as vandetanib or sunitinib have shown antineoplastic activity in ATC cells [96,97] and novel MKIs with antiangiogenic properties, CLM94, CLM3, CLM29 and CLM24, are active in ATC cells [98]. Clinical trials with lenvatinib or sorafenib have shown modest activity. Apart from dabrafenib and trametinib, other *BRAF* inhibitors have not yet been tested in clinical trials; conflicting results in the use of vemurafenib have been reported [99,100]. Furthermore, a recent report has suggested that the development of different acquired secondary *RAS* mutations by ctDNA, which was found in 4 patients with *BRAFV600E* mutated PTC and ATC treated with BRAF inhibitors, could be a potential resistance mechanism [101].

## 4. Future Landscape

### 4.1. RET (Rearranged during Transfection) Inhibition

#### 4.1.1. Differentiated Thyroid Cancer

Preclinical studies have showed promising results, with a ≥ 10-fold increased potency from selective RET inhibitors in comparison with MKI [45]. Pralsetinib (BLU-667) and selpercatinib (LOXO-292) are selective *RET* inhibitors that have shown activity against most common oncogenic RET alterations, including *RET* M918T and other gatekeeper mutations, such as V804M, often considered as primary resistance to MKI [102]. They directly inhibit *RET* autophosphorylation by competing with ATP for binding. Both are highly potent and selective *RET* inhibitors that have been tested in phase I/II clinical trials harboring advanced solid tumors with *RET* alterations [38,39]. The ARROW study has presented updated results in 11 patients with RET-fusion DTC treated with pralsetinib, reaching an impressive ORR of 91% and a DCR of 100% [38,103]. Additionally, the selpercatinib LIBRETTO-001 trial has also updated results in *RET*-fusion thyroid cancer, reaching an ORR of 79% and, again, a DCR of 100%, as well as a median duration of response of 18 months (95% CI: 7.6-NE). PFS was not reached (95% CI: 10-NE) [104,105]. These results have led the approval of selpercatinib for patients with advanced or metastatic *RET* fusion-positive RAI-DTC who require systemic therapy.

#### 4.1.2. Medullary Thyroid Cancer

*RET* inhibitors in MTC have also shown spectacular antitumor activity leading to the approval of selpercatinib by the FDA for the treatment of *RET*-mutated MTC [106]. This drug was analyzed in a cohort of *RET*-mutated MTC patients in the phase I/II LIBRETTO-001 trial, achieving an ORR of 69% with a DCR of 95% in pretreated patients and an ORR of 73% with a DCR of 95% in treatment naïve patients [104]. A phase III randomized trial is currently ongoing for *RET*-mutated MTC patients not previously treated (LIBRETTO-531; NCT04211337).

Preliminary results from pralsetinib have shown a 60% ORR and 18-month duration of response rate of 90% in previously treated *RET*-mutant MTC, as well as a 74% ORR in treatment-naïve *RET*-mutant MTC patients [107]. Other ongoing clinical trials featuring MKIs with *RET* inhibition activity are shown in Table 4.

### 4.2. Immunotherapy

It is well-known that neoplasms with a high tumor mutational burden (TMB) are tend to be responsive to immunotherapy. Despite the fact that thyroid cancer has a rather low TMB (around 0.4 mutations/Mb) [110], there is a rationale for the use of immunotherapy in these tumors [111].

#### 4.2.1. Differentiated Thyroid Cancer

The study of the tumor microenvironment (TME) showed that thyroid tumoral tissues overexpress CSF-1 and CCL-2 that attract tumor associated macrophages (TAMs). Aberrant tyrosine kinases have the ability to modulate the immune system and the tumor microenvironment (TME), and the use of TKI targeting VEGFR seems to increase PD-1 expression. This is highly important in ATC. However, high levels of TAMs have also been found in DTC. In the case of *BRAFV600E*-mutated DTC, PD-L1 overexpression is frequently found in comparison with *BRAF WT* tumors (53% vs. 12.5%) [112,113].

In this setting, different treatment approaches have been evaluated: the double combination of immune checkpoint inhibitors (ICIs) and the combination of ICIs with TKIs or chemotherapy. A phase Ib basket trial (Keynote-028) with pembrolizumab (anti-PD-L1) in advanced solid tumors showed mild activity in DTC, with an ORR of 2%, stabilization of the disease at 6 months in 69%, a clinical benefit rate of 50% and a median PFS of 7 months in the cohort of 22 DTC patients [114]. Further research is required and a phase II clinical trial is ongoing (NCT02628067). ICIs combinations, such as nivolumab and ipilimumab, ICIs and TKIs combination, such as pembrolizumab plus lenvatinib, have been analyzed in different thyroid cancer cohorts showing modest increase of antitumoral activity in DTC [115,116]. Other combinations are under research. There is an interesting ongoing phase II study that is enrolling PDTC and ATC patients to be treated with different targeted therapies or chemotherapy with atezolizumab, depending on the presence of RAS/BRAF mutations (NCT03181100). Triple therapy is being tested in a cabozantinib, nivolumab, and ipilimumab (CaboNivoIpi) clinical trial for advanced DTC (NCT03914300).

With regard to possible targets to TME, a CSF-1R inhibitor is being evaluated against advanced solid tumors in monotherapy (NCT01346358) or with paclitaxel (NCT01525602). Furthermore, the efficacy of CSF-1R in combination with pembrolizumab (NCT02452424) and tremelimumab or durvalumab in solid tumors (NCT02718911) is under clinical research.

#### 4.2.2. Medullary Thyroid Cancer

Until very recently, the immune microenvironment of MTC had not been studied. The first data in this regard come from a series of 16 MTC tumor samples, where only one expressed PD-L1 in tumor cells and two in immune cells [117]. A posterior analysis of a larger population, showed a PD-1 expression in immune cells and PD-L1 expression in tumor cells in 25% and 22% of the samples, respectively. PD-1 positivity was significantly correlated with distant metastasis at surgery, particularly when PD-1 and PD-L1 were co-expressed [118]. Immune infiltration occurs in 49% and 90% of primary and metastatic tumors, respectively, being TCD8+ cells the dominant T-cell subtype [119]. It also seems that PD-L1 positivity is associated with a larger tumor size, lymph node metastasis and advanced TNM staging [120]. Therefore, since PD-1/PD-L1 expression seems to confer a more aggressive clinical course, the use of immune checkpoint inhibitors in this subgroup of patients may have a drastic impact in the natural history of their disease.

Although it first seemed that the mismatch repair system is not altered in MTC [121], mutations in MSH2 and MSH6 have been recently reported [119]. These findings suggest the emerging importance on the assessment of mutation burden and microsatellite instability in this subset of tumors in order to properly select patients for immunotherapy treatment.

#### 4.2.3. Anaplastic Thyroid Cancer

Immunotherapy represents a promising approach in the management of ATC. Tumor associated macrophages (TAMs) in ATC belong to the M2 phenotype, which promote tumor cells proliferation [122]. A high expression of TAMs is associated with a worse prognosis; hence it has been considered as a prognostic biomarker [123]. Inhibiting TAMs recruitment is a novel therapeutic approach in ATC. CSF1 is a lineage regulator of macrophages and is highly expressed in metastatic ATC cell lines with regard to primary ATCs or PTC cell lines, suggesting CSF1 expression is associated with a higher grade of malignancy and may be a potential target [124]. Vascular endothelial growth factor A (VEGF-A) causes a massive infiltration of TAMs and is upregulated in ATC, which makes it a potential target for inhibiting TAMs recruitment [125]. Chemokine (C-C motif) ligand 2 (CCL2) attracts monocytes that can later migrate to the tumor environment and differentiate into TAMs. It seems that p53 binds to CCL2 [126] and it has been suggested that targeting CCL2 in *p53*-mutated ATC may be an interesting treatment approach [127].

Oncolytic virus (OV) vaccines are based on the replication of viruses in cancer cells in order to destroy them while leaving intact healthy cells. OV vaccines have shown activity in ATC cell lines [128,129] and there are currently several ongoing clinical trials in solid tumors.

CAR-T cells targeting intercellular adhesion molecule-1 (ICAM-1) have proven to be very effective in a mouse model of ATC [130], making adoptive cell transfer a promising treatment option.

When it comes to the use of checkpoint inhibitors, the immune microenvironment of ATC has been recently studied. PD-L1 expression in ATCs seems to be as high as 70–90% and anti-PD-L1 treatment in murine models reduces the tumor volume [131]. Interestingly, PD-L1 expression in ATC is associated with *BRAFV600E* mutation. This led to the combination of PD-L1 and *BRAF* inhibition in a murine model, showing again a shrinkage in tumor volume [132]. Various checkpoint inhibitors are currently under study in randomized clinical trials, summarized down below [133,134] (Table 5).

PD-1 inhibitor spartalizumab was the first ICI to show responsiveness in ATC, with an overall response rate of 19%, including three patients with a complete response and five with a partial response. In the PD-L1 positive population, the 1-year survival was 52.1% [133]. In addition, the combination of nivolumab and ipilimumab seems to be active in ATC with 3 out of 10 patients showing long-term major partial response in a phase II trial with 49 thyroid cancer patients [115].

The combination of atezolizumab with vemurafenib/cobimetinib (*BRAF*-mutated ATC), cobimetinib *(RAS, NF1* or *NF2*-mutated ATC), bevacizumab (none of these mutations) or paclitaxel (patients who did not qualify for the other three cohorts), has shown promising early results in the first two cohorts of patients with unreached OS in the vemurafenib/cobinetinib arm and a median OS of 18.23 months in the second cohort [134].

### 4.3. NTRK Inhibitors

Even though *NTRK* rearrangement is threefold less frequent than *RET* mutations, a prevalence of TRK fusions has been reported between 5–25% in thyroid cancer and may be seen in DTC, PDTC and ATC. If no other driver mutation is found, this may be a potential therapeutic target using first generation *NTRK* inhibitors, larotrectinib or entrectinib. The former is an inhibitor of tropomyosin receptor kinase (TRK) A (TRKA), TRKB, and TRKC, which has been evaluated in two phase I trials and one phase II basket trial, including 26 patients with thyroid cancer (22%). An initial outstanding ORR of 79% was found [136] and a recent update of these three trials showed a median duration of response of 35.2 months and a median PFS of 25.8 months, with most adverse effects being grade 1–2 [137,138,139]. Entrectinib also inhibits TRKA, TRKB, and TRKC but it blocks *ALK* and *ROS* too. In 2018, 54 patients with *NTRK*-fusion–positive solid tumors were evaluated, including 4 thyroid cancers, with an ORR of 57.4% and partial responses observed in all thyroid neoplasms included [137,140,141].

### 4.4. ALK (Anaplastic Lymphoma Kinase) Inhibitors

#### 4.4.1. Differentiated Thyroid Cancer

Anaplastic lymphoma kinase (*ALK*) is a kinase which activates MAPK and PI3K/AKT pathways. Its rearrangements, mainly *EML4-ALK* fusions, are found mostly in MTC and ATC. A whole-genome sequencing of PTC found translocation in 2.2% of PTCs, primarily *STRN-ALK* fusion, being associated to females, a younger age and an aggressive behavior. It is more prevalent in diffuse sclerosing variants of PTC and PDTC. In these cases, the combination of standard treatment with an *ALK* inhibitor such as crizotinib may be useful, but further studies are required [142,143].

#### 4.4.2. Medullary Thyroid Cancer

A Korean group sequenced 84 sporadic MTCs, discovering 101 hotspot mutations in 18 genes [144]. They evaluated *ALK* rearrangement through immunohistochemistry and fluorescence in-situ hybridization (FISH), finding two positive cases. For the first case a novel glutamine fructose-6-phosphate transaminase 1 (*GFPT1)-ALK* fusion was detected and the second case held the echinoderm microtubule-associated protein-like 4 *(EML4)-ALK* fusion, with breakpoints located in intron 13 of E*ML4* and intron 19 of *ALK*, which is the most common variant in non-small-cell lung cancer (NSCLC). In fact, this patient presented lung metastasis after total thyroidectomy and was enrolled in a phase I crizotinib trial, achieving partial response [145]. Recently, a novel CCDC6-ALK fusion was found in a 10 year old child with metastatic MTC that progressed to RET inhibitor selpercatinib, but rapidly responded when treatment was switched to *ALK* inhibitor alectinib [146]. This suggest that, although infrequent, *ALK* rearrangements do exist in MTC and respond to *ALK* targeted therapy.

#### 4.4.3. Anaplastic Thyroid Cancer

*ALK* genetic alterations have been described in ATC. Point mutations in the *ALK* gene appear in approximately 10% of ATCs according to available data [147]. Two-point mutations in exon 23 of the *ALK* gene, C3592T and G3602A, were associated with an increased tyrosine kinase activity and cell growth and invasion [148]. A fusion between *ALK* and striatin gene (STRN) has been reported in one patient with ATC [149], who was subsequently treated with crizotinib showing an impressive clinical response [150]. After 36 months of near complete response, the patient relapsed and a new tumor sample was obtained, finding the same *STRN/ALK* fusion. Treatment with a second-generation *ALK* inhibitor ceritinib resulted in a 16 months complete response. After brain progression, treatment with brigatinib rapidly led to partial response [151]. Again, although rare, *ALK*-mutant ATCs may be responsive to treatment with *ALK* inhibitors.

### 4.5. Radioactive Iodine Resensitization/Redifferentiation

Another approach to RAI refractoriness is to re-sensitize tumors to RAI. The sodium/iodide symporter (NIS) is a plasma membrane glycoprotein responsible for the iodide active transport in thyroid-follicular cells. In normal conditions, TSH stimulates its expression through cAMP pathway via PAX8. RAI therapy is based on its entrance through NIS, with emission of beta particles and destruction of the cell. Hence, NIS loss of expression is a mechanism of RAI resistance and, as we have already mentioned, 70% of DTC present a mutation in MAPK pathway, resulting in loss of expression of NIS. Because of this mechanism, the inhibition of *BRAF* activation by *MEK* or *BRAF* inhibitors, allows the expression of NIS, thus, resensitizing tumors to RAI. Another proposed mechanism is that inhibition of mTOR promotes redifferentiation through the stimulation of thyroid transcription factor-1 (TTF1), which enhances NIS expression. Nonetheless, further studies with regard to the use of mTOR inhibitors with this purpose are required [152].

Selumetinib, a *MEK1/2* inhibitor, is under investigation in a multicenter, phase-II trial with promising results based on the pilot study performed by Ho et al. where 12/20 (60%) participants increased radioiodine uptake, with 8/12 being able to receive RAI. 63% achieved partial responses and 37% stable disease as best radiological response [153,154].

Dabrafenib and Vemurafenib, both selective *BRAF* inhibitors, have been tested to determine whether they can increase RAI uptake in *BRAFV600E*-mutated DTC. With dabrafenib, 60% of the patients demonstrated new RAI uptake. They were treated with RAI afterwards, with 33% reaching partial responses and 66% stable disease. Vemurafenib was tested by Dunn et al. in *BRAFV600E*-mutated DTC, with 60% of sensitization to RAI, but only 40% have enough avidity to receive RAI. In this case, 50% reached partial response and 50% stable disease. This trial delineates the contribution of tumor response attributable to the use of a *BRAF* inhibitors and not only because of the re-use of RAI [155,156].

### 4.6. Other Novel Therapeutic Approaches

#### 4.6.1. Peptide Receptor Radionuclide Therapy (PRRT)

In PRRT, different radionuclides bind to cancerous cells expressing somatostatin receptors (SSTR), mainly SSTR2, allowing the internalization of the radionuclide that delivers radiation directly inside the tumor cell. Due to their neuroendocrine origin, MTC cells express SSTR2. Lutetium-177 (177Lu)-DOTATATE has shown promising results in a phase II clinical trial including SSTR2-expressing MTCs reaching a 40% of PR and SD and general improvement in quality of life. In the case of yttrium-90 (90Y)–DOTATOC, phase II trials have shown benefit in OS. PRRT has also been tested in DTC and ATC, with more modest results; SD of 29% within DTC patients and no responses among ATC patients [157,158].

High specific activity 131I metaiodobenzylguanidine (MIBG) targets the catecholamine transporter, being nowadays the standard of care for metastatic or unresectable paraganglioma and pheochromocytoma. In TC, only case series have been published concerning MTC, reporting some PR, mainly in the sporadic subgroup. Further studies with regard to this radio-labelled molecule are necessary [159,160].

#### 4.6.2. Epidermal Growth Factor Receptor 1 (EGFR) Epidermal Growth Factor Receptor 2/3 (HER2/3)

The addition of a HER2/HER3 kinase inhibitor enhances the effect of *BRAF* inhibitors by sensitizing thyroid cells to them. The reason is that *BRAF* inhibitors increase the transcription of HER3 expression, which dimerizes with HER2 by binding neuroregulin-1 and, hence, reactivates MAPK pathway, promoting resistance to BRAF inhibitors. A phase I is evaluating this combination in *BRAFV600E*-mutated DTC (90%) and ATC (10%), with a response rate of 58% and PFS of 18 months in the DTC subgroup (NCT01947023) [112,161,162].

The use of Neratinib, a pan-HER inhibitor, in combination with everolimus, palbociclib or trametinib is being evaluated in a phase I trial for refractory and advanced or metastatic solid tumors with *EGFR* mutation/amplification, *HER2* mutation/amplification, or *HER3/4* mutation or *KRAS* mutation, with no results posted to date (NCT03065387) [112,163].

#### 4.6.3. Mammalian Target of Rapamycin (mTOR)

The implication of the PI3K pathway in the resistance to conventional therapy is leading the research to the combination with mTOR inhibitors to overcome this resistance. The combination of sorafenib and everolimus versus sorafenib alone in patients with RAI hurthle cell thyroid cancer is under investigation in a phase II trial (NCT02143726) [112]. The role of mTOR inhibitors has also been analyzed in advanced MTC in a phase II trial with everolimus showing clinical benefit in those patients [164].

## 5. Conclusions

Thyroid cancer is a heterogenous disease, but recent advances in understanding its genomic landscape related to the different histology patterns have helped in the development of novel drugs. The first hit has been the approval of MKI that are able to inhibit different tyrosine kinases involved in the activation of key intracellular signalling pathways for thyroid cancer progression. Those drugs have reached phase III clinical trials, from the preclinical findings, with an increase in survival based on the results of phase III studies directed to patients suffering from RAI-DTC and MTC. The second hit has been the identification of selective targeted drugs with considerable antitumor activity and less toxicity, such as *RET* or *TRK* inhibitors. In light on the upcoming trials and approved drugs, it is required to perform tissue genotyping to patients needing systemic therapy for the identification of driver alterations and the selection of the adequate treatment. Also, germline testing is strongly recommended in case genetic counselling is required.

Additionally, new targets are now being explored in the field of thyroid cancer, such as MEK, ERBB2/HER2, ALK or SSTR in order to increase the therapeutic possibilities and offer the adequate directed treatment to the patients. Immunotherapy is also under research, but the efficacy of PD-1/PDL-1 or CTLA-4 inhibitors in all thyroid cancer subtypes is different and novel strategies in the immune cell cycle are needed to achieve better responses.

With these advances coming in, treatment options have significantly increased, but the optimal treatment sequence is still unknown. Though the first decision will be probably determined according to the genetic findings, phase III trials are currently analysing the best treatment option upfront. However, unfortunately, patients will eventually progress to these new drugs, so the identification of acquired resistance mechanisms is mandatory. In this setting, not only research based on tumor tissue, but also on liquid biopsy to identify driver mutations or resistance mechanisms in ctDNA, could be of great interest in order to direct the optimal sequence therapeutic algorithm with a more accessible sample.

In conclusion, thyroid cancer is a landmark in the development of new drugs based on genetic driver alterations. However, even though new drugs keep arising in the field, not all patients experiment antitumor response and most of them will eventually progress, many times showing an adequate performance status to continue receiving more therapeutic options. Thus, translational research focused on novel actionable targets and biomarkers selection is constantly needed.

## Figures and Tables

**Table 1:** Most relevant characteristics from phase III clinical trials in DTC.

	Sorafenib (DECISION) * [69]	Lenvatinib (SELECT) * [70]
**N**	417	392
**Target**	*RAF, VEGFR 1–3, PDGFR, c-KIT* and *RET*	*FGFR 1–4, VEGFR 1–3, RET, c-KIT* and *PDGFR α*
**Treatment line**	1st line	1st line or progressed to previous TKI (maximum one; 25%)
**Population**	RECIST Disease progression within the previous 14 months	IRR evidence of progression within the previous 13 months
**Histology**	Papillary (56.8%) Poorly differentiated (9.6%) Follicular (25.4%)	Papillary (50%) Poorly differentiated (10.7%) Follicular (20.3%) Hürthle cell (18.4%)
**Metastatic location**	Bone (27%) Only Lung (16.7%)	Bone (39.8%) Lung (86.6%)
**Comparator arm**	Placebo	Placebo
**Crossover placebo-intervention**	Allowed	Allowed
**Randomization**	1:1	2:1
**Primary Endpoint**	PFS	PFS
**Results**	10.8 vs. 5.8 months (*p* < 0.0001)	18.3 vs. 3.6 months (*p* < 0.001)
**Secondary Endpoints**	OS, TTP, DCR, ORR	ORR, OS
**Results**	OS: 39.4 vs. 42.8 months (52.8% vs. 54.8% deaths in 8 years) ^#^ ● Crossover correction: significance not reached	OS: 41.6 vs. 34.5 months ^+^ ● Subgroup analysis: improved OS in older population (>65 yo) ● Crossover correction: significance reached [71]
	TTP: 337 vs. 175 days (*p* <0.0001)	
	DCR: 86.2% vs. 74.6% (*p* = 0.015) ORR (No CR): 12.2% vs. 0.5% (*p* < 0.0001)	DCR: 87.7% vs. 55.7% (*p* < 0.001) ORR (4 CR): 64.8% vs. 1.5% (*p* < 0.001)
**Adverse Effects G3/G4**	37.2% (Hand-Foot syndrome)	75.9% (hypertension)
**Dose Reduction**	64.3%	67%
**Discontinuation**	19%	14%

PFS: progression free survival, OS: overall survival, TTP: time to progression, DCR: disease control rate, CR: complete response, ORR: objective response rate *: Both were double-blind, randomized, multicenter phase III trials. ^#^: Since the median value could not be estimated due to censored data, the percentage of participants who died is presented in original study. ^+^: The median OS was not reached in the original study at cut-off date for either the lenvatinib or the placebo arm (including crossover participants).

**Table 2:** Ongoing clinical trials with TKIs in DTC.

Drug	Combination	Drug Targets	Population	Phase	N	Primary Endpoint	Status	Results
Cabozantinib NCT03690388	N/A	*MET, VEGFR2, FLT3, c-KIT, and RET*	RAI-R DTC Progressed to 1 or 2 previous AntiVEGFR	III	300	PFS ORR	Recruiting	Not available
Donafenib NCT03602495	N/A	*RAF, VEGFR, PDGFR*	RAI-R DTC	III	204	PFS	Recruiting	Not available
Apatinib NCT03048877	N/A	*VEGFR2*	RAI-R DTC	III	118	PFS	Active, not recruiting	Not available
Axitinib NCT00389441	N/A	*VEGFR, PDGFR, c-kit*	RAI-R DTC Unresectable Locally-Advanced Thyroid Cancer	II	52	ORR	Completed	34.6% [73]
Axitinib NCT00094055	N/A	*VEGFR, PDGFR, c-kit*	RAI-R DTC Metastatic ATC, MTC	II	60	ORR	Completed	30% [74]
Motesanib NCT00121628	N/A	*VEGFR, PDGFR, c-kit*	RAI-R DTC MTC	II	184	ORR	Completed	14% [75]
Sulfatinib NCT02614495	N/A	*VEGFR, FGFR1*	RAI-R DTC MTC	II	66	ORR	Completed	Not available
Sunitinib NCT00519896	N/A	*PDGFR, FLT3, c-KIT, VEGFR, RET*	RAI-R DTC MTC	II	35	ORR	Completed	33.3% [76]
Pazopanib NCT01813136	N/A	*VEGFR, PDGFR, c-kit*	RAI-R DTC	II	168	TTF	Completed	Not available
Dovitinib NCT01964144	N/A	*VEGFR, FGFR*	RAI-R DTC MTC	II	40	ORR	Completed	Not available
Anlotinib NCT02586337	N/A	*VEGFR, FGFR, PDGFR, c-kit*	RAI-R DTC	II	113	PFS	Active, not recruiting	Not available
Sorafenib NCT00654238	N/A	*VEGFR, PDGFR, BRAF*	RAI-R DTC, ATC, MTC	II	59	ORR, SD	Completed	86.4% in DTC; 50% in PDTC [77]
Selumetinib NCT00559949	N/A	*MEK 1–2*	RAI-R DTC	II	39	ORR	Completed	3.1% [78]
Vemurafenib NCT01286753	N/A	*BRAF*	BRAF V600E RAI-R DTC	II	51	ORR	Completed	42.3% [72]
Dabrafenib NCT01947023	Lapatinib	*BRAF + EGFR/HER2*	BRAF V600E or V600K RAI-R DTC	I	21	MTD	Active, not recruiting	Not available
Dabrafenib NCT01723202	Trametinib	*BRAF +/- MEK*	BRAF + RAI-R DTC	II	53	ORR	Active, not recruiting	Not available
Trametinib NCT03244956	Dabrafenib	*MEK +/- BRAF*	RAS (NRAS or KRAS or HRAS) or BRAFV600E or K601E mutation RAI-R DTC	II	87	ORR	Recruiting	Not available
Everolimus NCT01164176	N/A	*mTOR*	RAI-R DTC	II	40	DCR	Completed	81% [79]
Everolimus NCT01263951	Sorafenib	*mTOR + VEGFR, PDGFR, BRAF*	RAI-R DTC progressed to sorafenib	II	35	PFS, ORR, SD	Completed	Not available
Temsirolimus NCT01025453	Sorafenib	*mTOR + VEGFR, PDGFR, BRAF*	RAI-R DTC	II	37	ORR	Completed	26.7% [80]
Everolimus NCT03139747	Lenvatinib	*mTOR + FGFR, VEGFR, RET, c-KIT and PDGFR α*	RAI-R DTC progressed to lenvatinib	II	5	PFS	Completed	Not available
Sorafenib NCT02143726	Everolimus	*VEGFR, PDGFR, BRAF +/− mTOR*	RAI hurthle cell thyroid cancer	II	35	PFS	Active, not recruiting	Not available
Neratinib NCT03065387	Everolimus, palbociclib or trametinib	*Pan-HER inhibitor + mTOR, CDK4/6, MEK*	Refractory and Advanced or Metastatic Solid Tumors	I	120	MTD	Recruiting	Not available

RAI-R DTC: radioiodine-refractory differentiated thyroid cancer, MTC: medullary thyroid cancer, ATC: anaplastic thyroid cancer, PFS: progression free survival, ORR: objective response rate, TTF: time to treatment failure, MTD: maximum tolerated dose, SD: stable disease, N/A: not/applicable.

**Table 3:** Main characteristics from the phase III trials in MTC with vandetanib and cabozantinib.

	ZETA [83]	EXAM [84]
Study design	Randomized (2:1), double blind, placebo-controlled, phase III	Randomized (2:1), double blind, placebo-controlled, phase III
Experimental arm	Vandetanib 300mg/24h	Cabozantinib 140 mg/24 h
Target	*VEGFR, EGFR, RET*	*MET, VEGFR2, FLT3, cKIT and RET*
Number of patients	331	330
Tumor stage	Unresectable/Metastatic	Unresectable/Metastatic Documented RECIST progression
Previous treatment lines	132 (40%) previously treated	128 (40%) previously treated
RET mutational status: RET+/RET-/RET unknown	56% (187)/2.4% (8)/41% (136)	48% (159)/12% (41)/39% (130)
Primary endpoint	PFS	PFS
Progressive disease	Not mandatory	Yes
Overall Response Rate	45% vs. 13%	28% vs. 0%
Disease Control Rate	87% vs. 71%	55.3% vs. 13.5%
Progression Free Survival (PFS)	30.5 m vs. 19.3 m (HR 0.27)	11.2 m vs. 4 m (HR 0.28)

PFS: progression free survival.

**Table 4:** Ongoing clinical trials involving MKIs with RET inhibition activity in MTC.

Drug	Combination	Drug Targets	Population	Phase	N	Primary Endpoint	Status	Results
Selpercatinib (LOXO-292) NCT03157128	N/A	*RET*	Advanced solid tumors	I/II	970	MTD, RP2D, ORR	Recruiting	56% [104]
Selpercatinib (LOXO-292) NCT04211337	Cabozantinib Vandetanib	*RET*	RET-m MTC	III	400	TFFS	Recruiting	Not available
Pralsetinib (BLU-667) NCT03037385	N/A	*RET*	Advanced solid tumors	I/II	527	MTD, ORR	Recruiting	89% [107]
Anlotinib NCT02586350	N/A	*VEGFR, FGFR, PDGFR, c-kit, RET*	MTC	IIB	91	PFS	Completed	20.67 months [108]
Surufatinib NCT02614495	N/A	*VEGFR, FGFR, RET*	RAI-R DTC, MTC	II	66	ORR	Completed	PR 17% [109]
Nintedanib NCT01788982	N/A	*VEGFR, FGFR, PDGFR, RET*	DTC, MTC	II	143	PFS	Active, not recruiting	Not available
Regorafenib NCT02657551	N/A	*VEGFR, PDGFR, c-kit, RET*	MTC	II	33	PFS	Recruiting	Not available
BOS172738 NCT03780517	N/A	*VEGFR, RET*	RET-gene altered tumors	I	144	TEAE, MTD, RP2D	Recruiting	Not available
TPX-0046 NCT04161391	N/A	*SRC, RET*	RET-gene altered tumors	I/II	362	DLTs, MTD, ORR	Recruiting	Not available

RAI-R DTC: radioiodine-refractory differentiated thyroid cancer, MTC: medullary thyroid cancer, MTD: maximum tolerated dose, RP2D: recommended phase 2 dose, ORR: objective response rate, PFS: progression free survival, TFFS: treatment failure free survival, TEAE: treatment emergent adverse event, DLTs: dose-limiting toxicities, N/A: not/applicable.

**Table 5:** Ongoing clinical trials with immunotherapy in ATC.

Target	Drug	Combination	Population	Phase	N	Primary Endpoint	Results
PD-1	Pembrolizumab NCT02688608	N/A	ATC	II	20	RR	Not available
	Pembrolizumab NCT03072160	N/A	MTC	II	17	CL ^#^, PR/CR	Not available
	Pembrolizumab NCT03012620	N/A	Rare cancers	II	350	ORR	Not available
	Pembrolizumab NCT02628067	N/A	Advanced solid tumors	II	1350	ORR	Not available
	Pembrolizumab NCT02054806	N/A	Advanced solid tumors	Ib	477	Best OR	ORR 9% [114]
	Pembrolizumab NCT03360890	Docetaxel	TC and salivary gland tumors	I	46	ORR	Not available
	Pembrolizumab NCT03211117	Docetaxel Doxorubicin	ATC	II	3	OSR	Terminated [135]
	Pembrolizumab NCT02973997	Lenvatinib	RAI-R DTC	II	60	CRR > 15%	Primary endpoint not reached [116]
	Pembrolizumab NCT04171622	Lenvatinib	ATC	II	25	OS	Not available
	Pembrolizumab NCT04234113	SO-C101	Solid Tumors	I/Ib	96	DLTs, AEs	Not available
	Nivolumab NCT04061980	Encorafenib Binimetinib	RAIR BRAF-mutated DTC	II	40	ORR	Not available
	Spartalizumab NCT02404441	N/A	Advanced solid tumors	I/II	319	RP2D, DLTs, ORR	19% * [133]
	Cemiplimab NCT04238624	DabrafenibTrametinib	BRAF V600E ATC	II	15	ORR	Not available
PD-1 and CTLA-4	Nivolumab Ipilimumab NCT03246958	N/A	RAI-R DTC, ATC, MTC	II	54	RR (CR + PR)	9.4% (DTC) 30% (ATC) 0% (MTC) [115]
	Nivolumab Ipilimumab NCT03914300	Cabozantinib	DTC	II	24	ORR	Not available
	Nivolumab Ipilimumab NCT02834013	N/A	Rare tumors	II	818	ORR	Not available
PD-L1	Atezolizumab NCT03181100	1: Vemurafenib/Cobimetinib 2: Cobimetinib 3: Bevacizumab 4: Paclitaxel	PDTC, ATC Cohort selection depending driver mutation	II	50	OS	1: not reached 2: 18.23 mo 3: 6.21 mo 4: 4.44 mo [134]
	Atezolizumab NCT03170960	Cabozantinib	Locally advanced or metastatic solid tumors	Ib	1732	MTD, ORR	Not available
	Atezolizumab NCT04400474	Cabozantinib	Advanced and progressive tumors from endocrine system	II	144	ORR	Not available
	Durvalumab NCT03215095	N/A	TC	I	11	DLTs	Not available
	Avelumab NCT03475953	Regorafenib	RAI-R DTC	I/II	362	RP2D, OR	Not available
PD-L1 and CTLA-4	Durvalumab Tremelimumab NCT03122496	SBRT	ATC	I	13	OS	Not available
	Durvalumab Tremelimumab NCT03753919	N/A	DTC, MTC, ATC	II	46	PFS, OS	Not available

TC: thyroid cancer, RAI-R DTC: radioiodine-refractory differentiated thyroid cancer, MTC: medullary thyroid cancer, PDTC: poorly differentiated thyroid cancer, ATC: anaplastic thyroid cancer, RR: response rate, CL: calcitonine levels, DLTs: dose-limiting toxicities, ORR: objective response rate, CRR: complete response rate, OS: overall survival, AEs: adverse events, MTD: maximum tolerated dose, RP2D: recommended phase 2 dose, PFS: progression free survival, CR: complete response, PR: partial response, mo: months. * ORR in the ATC cohort. ^#^ 50% or greater decline in calcitonin levels, N/A: not/applicable.
